# Chewing-gum stimulation did not reduce the absorbed dose to salivary glands during radioiodine treatment of thyroid cancer as inferred from pre-therapy ^124^I PET/CT imaging

**DOI:** 10.1186/s40658-014-0100-1

**Published:** 2014-12-06

**Authors:** Walter Jentzen, Marion Richter, James Nagarajah, Thorsten Dirk Poeppel, Wolfgang Brandau, Colin Dawes, Andreas Bockisch, Ina Binse

**Affiliations:** Klinik für Nuklearmedizin, Universität Duisburg-Essen, Hufelandstrasse 55, D-45122 Essen, Germany; Department of Oral Biology, Faculty of Dentistry, University of Manitoba, 780 Bannatyne Avenue, Winnipeg, Manitoba,, R3E 0W2 Canada

**Keywords:** Salivary gland, Thyroid carcinoma, Radioiodine therapy, Dosimetry, Iodine-124

## Abstract

**Background:**

The goal of this prospective study was to estimate the absorbed (radiation) doses to salivary glands in radioiodine therapy of thyroid cancer under chewing-gum stimulation using ^124^I positron emission tomography (PET)/computed tomography (CT) imaging.

**Methods:**

Duplex ultrasonography was conducted in three test persons for visual comparison of the glandular blood flow with three different stimulation types (no stimulation, chewing tasteless gum base, sucking on lemon slices). Ten patients with newly diagnosed differentiated thyroid cancer received ^124^I PET/CT dosimetry after thyroidectomy and prior to radioiodine therapy. Patients underwent a series of three ^124^I PET/CT scans (4, 24, and ≥96 h after administration of 23 MBq ^124^I). They were instructed to chew gum base (tasteless) approximately 20 min after ingesting the ^124^I-containing capsule in the course of the first day. Absorbed doses per administered ^131^I activity to the salivary glands were calculated and compared with the previously published results of the lemon-juice stimulation and non-stimulation groups.

**Results:**

The sonograms in the three test persons showed that glandular blood perfusion by lemon-juice stimulation was clearly increased compared with non-stimulation or chewing of gum base. The sonogram comparison between the chewing-gum stimulation and non-stimulation demonstrated a minor increase of blood flow for the gum base-stimulated salivary glands. The mean ± standard deviation of the absorbed dose per activity under chewing-gum stimulation for the submandibular and parotid glands (within parentheses) was 0.22 ± 0.09 Gy/GBq (0.22 ± 0.08 Gy/GBq). Compared with the absorbed doses of the non-stimulation group, 0.24 ± 0.08 Gy/GBq (0.21 ± 0.05 Gy/GBq), those of the chewing-gum stimulation group showed no significant change (*P* > 0.60), but the absorbed doses of the lemon-juice stimulation group, 0.35 ± 0.14 Gy/GBq (0.33 ± 0.09 Gy/GBq), were significantly higher (*P* < 0.04) than those of the chewing-gum stimulation group.

**Conclusions:**

The results suggest that salivary flow induced by chewing gum base does not cause a significant reduction of the salivary gland absorbed dose compared with that in the non-stimulation group. The increased blood flow appears to be a decisive factor causing the increased ^131^I absorbed doses in the lemon-juice stimulation group.

## Background

Radioiodine treatment of differentiated thyroid cancer has been proven highly effective; however, application of high ^131^I activity has been found to be associated with side effects. Organs at risk in high-activity radioiodine therapies are the bone marrow and the lung [1,2]. In addition, the parotid and submandibular glands may also be affected by high-activity radioiodine therapies, which may cause sialadenitis and xerostomia [3]. The radioiodine uptake in (major) salivary glands is mediated by the sodium-iodide symporter [4]. Sialadenitis appears transiently, but in particular, xerostomia can persist for an entire lifetime and may severely affect the patient's quality of life. For more than almost 30 years, radioprotective procedures have been proposed to mitigate damage to the salivary glands [5]. The use of sialogogic agents such as lemon juice, sour candies, and flavoured chewing gums is recommended [1,5]. The reasoning is based on the grounds that salivary gland stimulation after ^131^I administration reduces the radiation exposure time (residence time). Hence, radiogenic damage is thought to be reduced by increase of salivary flow.

Recent studies [6,7] by means of ^131^I scintigraphy supported the hypothesis. A case report [6] measured the ^131^I activity within the salivary gland before and shortly after lemon-juice stimulation. Scintigraphy images were acquired starting at 2 h after radioiodine administration. Single addition of lemon juice in the oral cavity produced a rapid decrease of approximately 80% to 90% of its initial value, but the ^131^I activity reached a maximum of its initial value after 10 to 30 min. In a follow-up study [7], similar results using ^123^I were obtained in 23 patients with the same methodology. Thus, the authors postulated that a repeated stimulation by lemon juice during radioiodine therapy should reduce the radioiodine residence time and consequently also the radiogenic damage. The reduction of the residence time of radioiodine (efflux > influx) within the salivary glands using sialogogic agents is termed the washout effect.

Three studies attempted to verify the proposed washout effect [8-10] using different methodologies under clinical conditions; however, none of these clinical studies could confirm the washout effect. In particular, Nakada et al. [8] observed in a prospective and longitudinal comparison study that an early start of sucking lemon candies (<1 h after ^131^I administration) induced a significantly higher incidence of sialoadenitis and xerostomia frequency compared with a 24-h delayed stimulation. Liu et al. [9] and our group [10] used a dosimetry approach. The absorbed (radiation) dose is an established quantity that is used to assess the potential for mediating the radiogenic damage. Liu et al. estimated the absorbed doses to the salivary gland using ^131^I scintigraphy. They compared the absorbed doses per administered ^131^I activity of four groups, who were stimulated at different time points after radioiodine administration (1, 5, 13, and 25 h) using vitamin C (as a substitute for lemon juice). No significant differences among the four groups were found. The authors concluded that salivary gland stimulation with vitamin C caused only a minor reduction of the absorbed dose. Our group estimated the absorbed doses using a more precise dosimetry approach than that used by Liu et al. [9]. We applied both positron emission tomography (PET) with ^124^I as a diagnostic equivalent to ^131^I scintigraphy and computed tomography (CT). The absorbed doses of two stimulation groups, each containing ten patients, were compared. The salivary glands of the first group were stimulated by chewing lemon slices shortly after ^124^I administration, whereas the second group was not stimulated. Against prevailing expectations, the ^124^I PET/CT salivary gland dosimetry yielded a significant increase of the absorbed dose per activity of 20% to 30% for the lemon-juice stimulation group compared with the non-stimulation group. The results of the three groups obviously contradict the prevailing opinion. A possible mechanism for explaining their results is the rebound effect. The sialogogic agents increase not only the salivary flow (efflux of radioiodine) but also the blood flow (influx of radioiodine). This combination may result in a net increase of the residence time of radioiodine (efflux < influx).

The present ^124^I PET/CT study further explored the radioiodine uptake mechanism of the salivary glands using another stimulation type. The aims of this prospective study were to assess the effect of chewing-gum stimulation on the absorbed doses to the salivary glands using ^124^I PET/CT and to compare the results with those for the non-stimulation and lemon-juice stimulation groups already reported [10].

## Methods

### Blood flow measurements

To determine the blood flow within the glandular tissue, an established methodology was applied [11] on three healthy test persons, two males of ages 38 and 47 and one female of age 23. Duplex sonography was conducted on these three subjects using an iU22 xMATRIX ultrasound system (Philips Healthcare, Hamburg, Germany). Initially, the salivary glands were imaged without stimulation and thereafter stimulated by either chewing gum base or sucking on lemon juice. The submandibular gland's sonograms were used representatively because they allowed a better visualization with ultrasound than the parotid glands [11]. Duplex ultrasonography was performed during sucking on a slice of lemon. In contrast, since imaging was difficult to perform during lower jaw movements, the images were acquired shortly after a period of 1 min of chewing-gum stimulation. For the comparison of the different stimulation types, the sonograms of the non-stimulated glands served as the reference standard.

### Patients

Our department has routinely been conducting pre-therapy ^124^I PET/CT scans for high-risk patients with differentiated thyroid cancer to perform lesion and organ-at-risk dosimetries [12]. Ten thyroidectomized patients prior to their first radioiodine therapy with histologically confirmed advanced differentiated thyroid cancer were included. Patients were not included if they had a medical history of salivary gland disease or affection, had received external beam radiotherapy to the neck or head, were taking anticholinergic medication, or had dental implants. Furthermore, patients bearing iodine-avid lymph node metastases near the salivary glands or thyroid remnant tissue with high uptake were not included, to avoid impairments in the absorbed dose calculations [13]. The standard patient preparation before ^124^I administration has been described previously [14]. Thyroid-stimulating hormone (TSH) stimulation was achieved by withdrawal of thyroid hormone. TSH levels before imaging were ≥25 mIU/mL. The mean ± standard deviation (SD) of the ^124^I activity was 22.7 ± 1.0 MBq. The patients gave written informed consent to perform the examination and the study was conducted in full accordance with regional ethical committee standard.

### Chewing-gum stimulation protocol

During the first day, the patients were instructed to chew on tasteless gum base (Solsona; Cafosa Gum S.A., Barcelona, Spain) approximately 20 min after capsule ingestion and to drink water. The patients were instructed to continue gum chewing with a frequency of 3 to 4 gums per hour and to drink at least 2 L of water. The patients ate lunch after the first 4-h ^124^I PET/CT investigation and later ate a snack and dinner. The intake of sour foods or drinks such as orange juice was avoided. The patients documented the time points of gum and water intakes during the first day.

### Salivary gland dosimetry protocol

The patients underwent a series of three ^124^I PET/CT scans on a Biograph Duo system (Siemens Medical Solutions, Chicago, IL, USA). PET imaging was acquired at about 4, 24, and ≥96 h after oral intake of a capsule containing [^124^I]NaI. The emission time was 300 s per bed position. The low-dose CT acquisition parameters were as follows: tube current time product of 15 mAs, tube voltage of 110 kVp, a pitch of 1.6, and a slice thickness of 5 mm. The emission images were reconstructed using the iterative attenuation-weighted ordered-subset expectation maximization algorithm with 4 iterations and 16 subsets. A post-reconstruction three-dimensional Gaussian smoothing filter of 5 mm was applied. Standard scatter, attenuation, and dead-time corrections provided by the manufacturer were used. The reconstructed transverse emission images had a voxel size of 1.7 × 1.7 × 2.4 mm^3^. The measured reconstructed PET spatial resolution (expressed as full width at half maximum) was 8.2 mm. The CT images were reconstructed using a reconstruction interval of 2.4 mm; the reconstructed image had a voxel size of 1.0 × 1.0 × 2.4 mm^3^.

### Patient analyses

The details of the patient analyses have been described in the literature [14]. The analyses consisted of image co-registration, delineation of the salivary gland volume, applying volume-dependent isovolume ^124^I recovery coefficients, and determination of the glandular residence times and absorbed doses per administered activity. Image co-registration across time was performed by matching the reference 4-h CT image with the 24-h CT image and the last CT image at ≥96 h. The resulting transformation parameters were then applied to the respective PET image for each time point. The salivary gland volumes were determined from the anatomical images by drawing a volume of interest around each salivary gland. The individual volume of interest was projected onto the co-registered PET image and used to determine the ratio of total activity to the gland volume; this ratio is referred to as the (imaged) isovolume activity concentration. The isovolume activity concentrations were then corrected for partial volume effect using isovolume ^124^I recovery coefficients derived from measurements with a phantom containing spheres of different volumes. It has been shown that the isovolume ^124^I recovery coefficients of spheres matched well with spheroids mimicking salivary glands. Finally, the radioactive decay law was applied to project the ^124^I activity concentration to the therapy nuclide. The ^124^I and projected ^131^I uptake values, *U*^124^(*t*) and *U*^131^(*t*) at the time point *t*, respectively, were determined.

The absorbed doses per ^131^I activity were estimated using a three-point model consisting of two contributions. The uptake curve was parameterized using a combination of linear functions from 0 to 24 h (first contribution) and a mono-exponential function from 24 h to infinity (second contribution). In the first part, trapezoid integration was applied for the area under the curve from zero time, *U*^131^(0) = 0, to 24 h, *U*^131^(24 h). The contribution resulting from the exponential phase was the time integral from 24 h to infinity using an effective ^131^I half-life that was calculated from the uptake values *U*^131^(24 h) and *U*^131^(≥96 h) by linear regression analysis. In the estimation of the glandular absorbed dose per activity, the volume, the residence time (see above), and the glandular tissue density were required. A density of 1.05 g/mL was used for the salivary glands. A uniform activity distribution within the salivary gland was assumed.

In the previous studies [10,14], the estimation of the absorbed doses per activity for the non-stimulation and lemon-juice stimulation groups was calculated using a seven-point model. In particular, additional PET measurements were acquired at 0.5, 1, 2, and 48 h after ^124^I administration, which were taken into account in the estimation of the residence time. The area under the uptake curve was determined by applying the trapezoid integration rule with a truncation at the time point of the last PET scan (≥96 h). To examine the effect of the reduced number of time points (three points vs. seven points) and the different integration approaches (piecewise trapezoid-exponential integration vs. trapezoid integration), the glandular absorbed doses per activity for the non-stimulation and lemon-juice stimulation groups were re-assessed using the three-point model and compared to those of the seven-point model.

### Statistical analysis

The descriptive statistics included the mean, the median, the SD, the minimum, and the maximum, which are provided in the following form: mean (median) ± SD (minimum to maximum). Differences among the groups were evaluated by the Mann-Whitney *U* test (non-parametric test). The correlation between the different quantities was evaluated using Spearman's rank correlation coefficient. A significance level (*P* value) of less than 5% was considered statistically significant.

## Results

### Blood flow measurements under three different stimulation types

**Figure 1:**
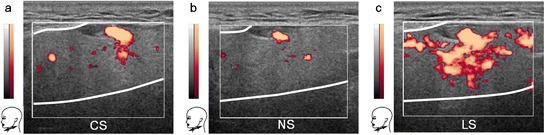
**Duplex sonograms of the salivary glands under different stimulation types.** The sonograms of a healthy person (male, 47 years) show relative changes in blood perfusion of the submandibular gland induced by chewing-gum stimulation **(a)**, non-stimulation **(b)**, and lemon-juice stimulation **(c)**. Blood flow information is superimposed in colour onto the conventional anatomical gray-scale image. The thick white lines within the thin white rectangles indicate the approximate glandular boundaries. *The chewing-gum stimulation, non-stimulation, and lemon-juice stimulation are designated by CS, NS, and LS, respectively*.

The qualitative analysis of the glandular duplex sonograms of the three test persons showed qualitatively different levels of blood perfusion within the glandular tissue (Figure 1). With reference to the sonograms obtained from the non-stimulated salivary gland, the blood perfusion was clearly increased under lemon-juice stimulation. In contrast, under chewing-gum stimulation, the glandular tissue exhibited a slight increase, which was irrelevant compared with the increase in level of blood perfusion under lemon-juice stimulation.

### Patient and stimulation characteristics of the chewing-gum stimulation group

The patient characteristics of the chewing-gum stimulation group are given in Table 1. In comparison with those of the non-stimulation and lemon-juice stimulation groups published previously [10], no significant differences among the groups were found except for the age. The patients in the lemon-juice stimulation group (*P* = 0.03) and the non-stimulation group (*P* = 0.10) were older, 51 (48) ± 14 years (31 to 73 years) and 48 (48) ± 18 years (22 to 72 years), respectively, than the patients in the chewing-gum stimulation group, 35 (30) ± 14 years (21 to 64 years). This age difference was a consequence of excluding patients with dental implants in this study.

**Table 1:** **Patient characteristics of the chewing-gum stimulation group**

**Characteristics**	**Chewing-gum stimulation group**
Started chewing gum base^a^	Approximately 20 min
Number of patients	10
Age (years)^b^	35 (30) ± 14 (21 to 64)
Gender (male/female)	4/6
Weight (kg)^b^	76 (72) ± 15 (59 to 104)
Histology (papillary/follicular)	8/2
TSH stimulation (endogenous/exogenous)^c^	10/0
TSH (mIU/L)^b^	74 (68) ± 35 (36 to 150)

^a^After ^124^I administration. ^b^Mean (median) ± SD (range). ^c^Thyroid-stimulating hormone (TSH) stimulation was achieved by hormone withdrawal (endogenous) or by injection of recombinant human TSH (exogenous).

The statistics of the number of chewing gums that were taken during the course of the first day were 6 (6) ± 2 (3 to 11) within the first 4-h interval (or before the first ^124^I PET/CT acquisition) and 13 (12) ± 7 (6 to 25) till the end of the first day. The water consumption ranged from 1.5 to 3 L with a mean (median) of 2.0 (2.0) L.

### Absorbed dose, glandular volume, and residence time of the salivary gland under chewing-gum stimulation

**Figure 2:**
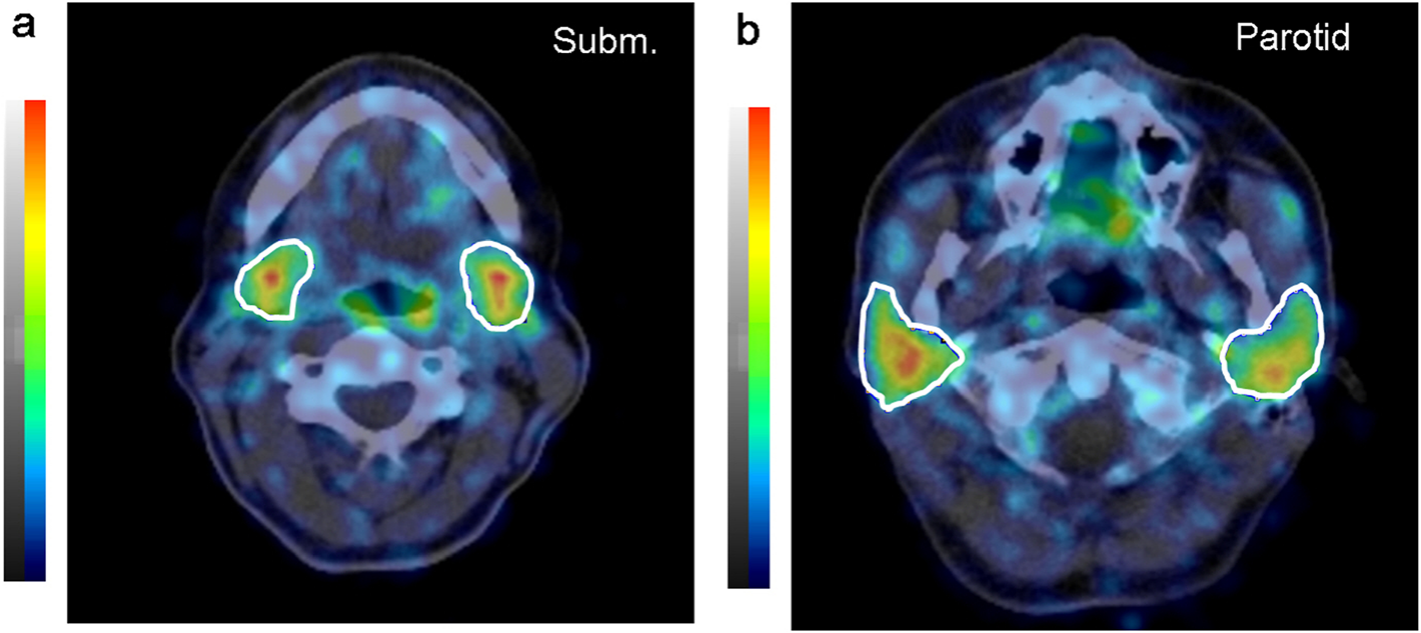
**Fused** ^**124**^ **I PET/CT slice views of the salivary glands 4 h after** ^**124**^ **I administration.** Transverse slice images of the submandibular **(a)** and parotid **(b)** glands with volumes of interest (indicated by white lines) are shown. The grayscale (CT) and colour scale (PET) ranges were −125 to 225 HU and 0 to 2 kBq/mL, respectively.

**Figure 3:**
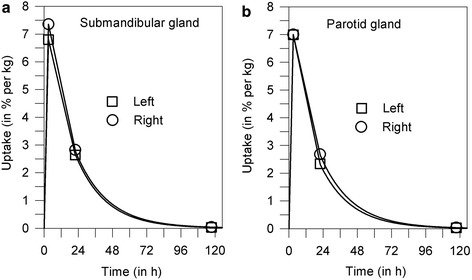
**Representative examples of the (projected)** ^**131**^ **I uptake curve of the salivary gland.** The measured uptake values (symbols) of the submandibular **(a)** and parotid **(b)** glands are in percentage of administered activity per kilogram of gland tissue. Solid lines indicate the fitted curves.

Figure 2 illustrates, as an example, fused PET/CT images of the submandibular and parotid glands with volumes of interest delineating the CT-based boundaries of the salivary glands. Representative examples of the uptake curves of the salivary glands are shown in Figure 3. The within-group statistical analysis revealed no statistically significant difference in the absorbed doses between the submandibular and parotid glands under chewing-gum stimulation (Table 2, *P* = 0.84). The mean ± SD absorbed doses to the salivary glands per activity was 0.22 ± 0.09Gy/GBq. The statistics of the single glandular volume were 9.53 (9.46) ± 1.59 mL (6.94 to 12.60 mL) for the submandibular glands and 20.90 (19.42) ± 6.13 mL (12.73 to 32.54 mL) for the parotid glands. The statistics for the residence time of the single submandibular and parotid glands were 1.10 (1.05) ± 0.40 min (0.51 to 2.08 min) and 2.35 (2.16) ± 1.07 min (1.20 to 4.70 min), respectively. The difference in the mean residence time between the gland types was attributed to the difference in gland volumes.

**Table 2:** **Absorbed dose per activity (in Gy/GBq) for the submandibular and parotid glands under different stimulations**

**Gland type**	**Statistics**	**Stimulation group**		
		**Chewing-gum**	**Non-stimulation** ^**a**^	**Lemon-juice** ^**a**^
Submandibular gland	Mean (median)	0.22 (0.22)	0.24 (0.22)	0.35 (0.29)
	±SD (min to max)	0.09 (0.09 to 0.36)	0.08 (0.14 to 0.45)	0.14 (0.20 to 0.67)
	Deviation (%)^b^	−37 (0.04)	−31 (0.09)	Reference
Parotid gland	Mean (median)	0.22 (0.20)	0.21 (0.19)	0.33 (0.32)
	±SD (min to max)	0.08 (0.07 to 0.44)	0.05 (0.16 to 0.31)	0.09 (0.15 to 0.48)
	Deviation (%)^b^	−33 (<0.001)	−36 (<0.001)	Reference

^a^Data were taken from reference [10] and used to re-assess the absorbed doses using the three-point model. ^b^Percentage deviation from the mean of the lemon-juice stimulation group (reference) and its significant difference level (within parentheses).

Of note, the relationship between absorbed doses per ^131^I activity and the number of chewing gums yielded no correlations. Specifically, Spearman's rank correlation coefficients and *P* values (within parentheses) were −0.40 (0.02) for the number of chewing gums within the 4-h interval, −0.20 (0.25) for the number of chewing gums after the first PET/CT scan, and −0.24 (0.17) for the total number of gums chewed on the first day.

### Absorbed dose comparison of the three different stimulation types

**Figure 4:**
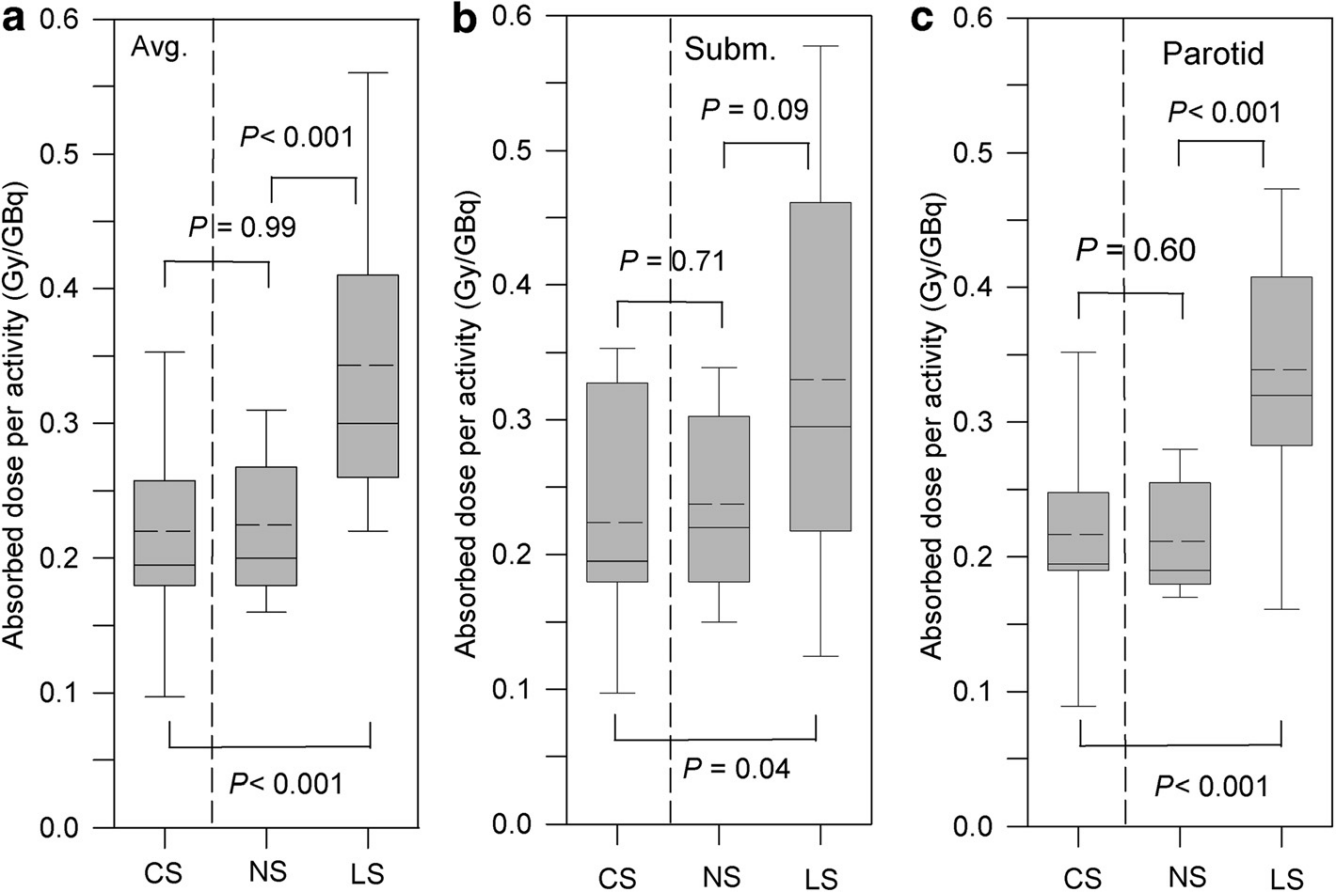
**Distribution of the absorbed dose per administered activity for the different stimulation types.** The absorbed dose distributions averaged over both gland types **(a)** and separately for the submandibular **(b)** and parotid glands **(c)** are shown. In the absorbed dose calculation, the 3-point model was used. The levels of significance between the groups are characterized by *P* values. *The chewing-gum stimulation, non-stimulation, and lemon-juice stimulation groups are designated by CS, NS, and LS, respectively*.

For all different stimulation groups, Table 2 juxtaposes the statistics of the absorbed doses to the submandibular and parotid glands. Figure 4 illustrates the distribution of the absorbed doses per activity. The absorbed doses of the non-stimulation and lemon-juice stimulation groups are shown to the right of the dashed horizontal lines in Figure 4; these absorbed doses were re-assessed using the three-point model. The absorbed doses per activity and the significant difference between non-stimulation and lemon-juice stimulation were similar to the results that were obtained using the seven-point model [10]. For example, the inter-group comparison revealed that the absorbed dose averaged over both gland types (Figure 4a) was significantly reduced by 32% (*P* < 0.001) in the non-stimulation group (0.23 Gy/GBq) compared with the lemon-juice stimulation group (0.34 Gy/GBq). The respective values using the seven-point model were 28% for the percentage decrease (*P* = 0.01) and 0.23 Gy/Gbq for the non-stimulation group and 0.32 Gy/GBq for the lemon-juice stimulation group. Hence, in all of the subsequent sections, the results of the three-point model are presented for the non-stimulation and lemon-juice stimulation groups and used for further analyses.

The inter-group comparison showed that the mean absorbed doses averaged over both gland types in the chewing-gum stimulation group were reduced by approximately 35% (0.22 Gy/GBq) compared with those in the lemon-juice stimulation group; this observed difference was statistically significant (*P* < 0.001, Figure 4a). The inter-group comparison, however, yielded no significant difference in the absorbed doses between the chewing-gum stimulation and non-stimulation groups (*P* = 0.99, Figure 4a). Separate inter-group comparisons for the parotid or submandibular glands exhibited a significant difference in the absorbed doses between the chewing-gum and lemon-juice stimulation groups for both the submandibular (*P* = 0.04, Figure 4b) and parotid glands (*P* < 0.001, Figure 4c).

## Discussion

Duplex sonography images were acquired for the submandibular glands under three different stimulation types (Figure 1). Using the non-stimulation images as reference, a minor increase of blood flow was observed under chewing-gum stimulation but a notable increase of the blood flow was seen under lemon-juice stimulation. The observed increase of blood flow for the lemon-juice stimulation compared with the non-stimulation was in agreement with the results of a study that used a similar methodology [11].

This finding allows the following conjecture: If the lemon-juice stimulation is actually associated with an increase of the absorbed dose per activity primarily caused by an increased glandular blood flow, the results of the sonography investigations suggest that the absorbed doses should be lower for the non-stimulation and chewing-gum stimulation groups. In addition, because Dawes and Macpherson [15] demonstrated that the salivary flow was higher while chewing gum base than for non-stimulation, it is expected that the absorbed doses will be lower for the chewing-gum stimulation group than for the non-stimulation and lemon-juice stimulation groups. Thus, the washout effect may dominate over the rebound effect when chewing gum base. These suggestions were partially confirmed in this study.

In line with the expectation, the comparison of the absorbed doses per activity between the lemon-juice and chewing-gum stimulation groups yielded, on average, a significantly lower value for the chewing-gum than for the lemon-juice stimulation group (Table 2 and Figure 4). The absorbed dose per activity was approximately 30% to 40% lower when chewing tasteless gum. The above findings demonstrate that the increased blood flow appears to be a decisive factor causing the increased absorbed doses in the lemon-juice stimulation group. However, no significant difference in the absorbed doses per activity (*P* ≥ 0.60) was observed between the chewing-gum stimulation and non-stimulation groups (Figure 4); the percentage difference of the mean absorbed dose was −8% for the submandibular gland and 5% for the parotid gland. Contrary to the expectation, chewing-gum stimulation and non-stimulation yielded similar absorbed doses per activity. Thus, the assumed washout effect was not large enough to alter significantly the absorbed dose between the chewing-gum stimulation and non-stimulation groups.

A reason for the statistically insignificant difference may be associated with a diminished washout effect by opposing changes. In detail, the non-stimulated salivary glands at rest exhibit a basic blood circulation and a basic salivary flow. It has been demonstrated that continuous chewing on tasteless gum base increases the salivary flow rate by a factor of approximately 3 (after 5 min) compared with the salivary flow rate at rest [15]; however, a slight increase in glandular blood flow is also observed as demonstrated by sonograms in this study. Hence, it appears that the efflux of radioiodine (increased salivary flow) is, to some extent, compensated for by a simultaneous influx of radioiodine (increased blood flow), which explains why the washout effect did not overly dominate under chewing-gum stimulation.

In addition, stimulation protocol-related and methodological factors may influence the outcome of the absorbed dose calculations. Variations of the number of chewing gums per hour and of chewing frequency may affect the results. Dawes and Macpherson [15] demonstrated that the flow rate ratio of initial to later chewing on gum base is approximately 5. Thus, the number of chewing gums on the first day influences the salivary flow rate; however, no correlation between the number of chewing gums and the absorbed doses per activity was observed in this study. Moreover, the salivary flow rate has been demonstrated to be independent of the chewing frequency after a few minutes [16]. Methodologically, the similar results of the absorbed doses using the three-point and seven-point models show that the outcome is not strongly affected by the calculation models. Moreover, the errors in the co-registration and the drawing of volumes of interest around the salivary glands have been shown to produce an average error of 9% in the absorbed dose calculation [17]. As a result, the factors mentioned are unlikely reasons for the unexpected findings.

Taken together, on the one hand, the use of chewing gums did not significantly decrease the salivary gland absorbed dose in radioiodine therapy and is therefore not helpful. Thus, the washout effect has not been proven, but neither can it be refuted. On the other hand, lemon-juice stimulation also did not reduce the absorbed dose as demonstrated by other studies [9,10]. The repeated use of lemon juice (every 15 to 20 min), which has been proposed by van Nostrand et al. [7] to mitigate the salivary gland damage, has not been proven to be favourable under clinical conditions and is possibly not accepted by the patients. The results suggest that the salivary glands should not be stimulated by either lemon juice or chewing gums.

## Conclusions

This study provided evidence, on the basis of ^124^I PET/CT imaging, that chewing-gum stimulation immediately after ^131^I administration did not significantly decrease the absorbed dose to the salivary glands in radioiodine therapy compared with the non-stimulated salivary glands. We suggest that the increased glandular blood flow overcompensates the washout effect in the lemon-juice stimulation group and, to a lesser extent, also in the chewing-gum stimulation group.
